# Interwoven Pathologies: The Complex Interplay of Social Anxiety Disorder, Body Dysmorphic Disorder, and Neuropathic Pain Post-Surgical Intervention

**DOI:** 10.1192/j.eurpsy.2025.1033

**Published:** 2025-08-26

**Authors:** V. H. Santos, F. M. Santos, P. Coelho, R. P. Andrade, I. A. Silva, T. Carvalhão, S. Fontes

**Affiliations:** 1 Department of Psychiatry and Mental Health, Cova da Beira Local Health Unit; 2 Faculty of Health Sciences, University of Beira Interior, Covilhã; 3 Department of Psychiatry and Mental Health, Médio Tejo Local Health Unit, Tomar; 4 Department of Psychiatry and Mental Health, Viseu Dão-Lafões Local Health Unit, Viseu; 5 Department of Psychiatry and Mental Health, Norte Alentejano Local Health Unit, Portalegre, Portugal

## Abstract

**Introduction:**

Social anxiety disorder (SAD) and body dysmorphic disorder (BDD) share a unique and intricate relationship, often presenting with overlapping yet distinct anxieties about social evaluation. In patients who have undergone repeated surgical interventions, the added dimension of neuropathic pain further complicates the clinical picture, as seen in this 48-year-old male. His preoccupation with his perceived nasal and upper lip deformities, coupled with neuropathic pain from multiple reconstructive surgeries, underscored a profound interplay of psychological and somatic symptoms, warranting a multidimensional therapeutic approach.

**Objectives:**

To elucidate the diagnostic overlap between SAD and BDD in a patient with neuropathic pain post-surgery, to explore the off-label use of pregabalin in addressing both anxiety and neuropathic pain symptoms, and to highlight the subtle clinical distinctions between social phobia related to perceived physical flaws (BDD) versus the broader fear of negative evaluation in social contexts (SAD).

**Methods:**

The patient’s history includes five reconstructive surgeries to correct perceived nasal and upper lip deformities, which resulted in significant spastic neuropathic pain. This pain was exacerbated by the patient’s intense anxiety regarding his physical appearance, manifesting as avoidance behaviors, fear of negative social evaluation, and repetitive self-evaluative behaviors typical of BDD. Initial treatment involved clonazepam and mirtazapine combined with psychotherapy. Despite partial relief of anxiety symptoms, neuropathic pain persisted, exacerbating his body image concerns. Pregabalin was introduced under the assumption that neuropathic pain was worsening his BDD-related distress and social anxiety symptoms.

**Results:**

Following the introduction of pregabalin, the patient demonstrated marked clinical improvements. The dual efficacy of pregabalin, particularly in its off-label role for social anxiety, contributed to a reduction in the fear of social situations, previously fueled by BDD-related preoccupations. The remission of neuropathic pain, in conjunction with improved social functioning, underscores the utility of pregabalin in addressing the complex somatopsychic interactions in patients with comorbid psychiatric and neuropathic conditions.

**Conclusions:**

This case highlights the intricate diagnostic and therapeutic complexities in managing co-occurring social anxiety and body dysmorphic disorder, where psychosomatic pain further distorts the clinical picture. The addition of pregabalin, despite its off-label status for SAD, proved to be a pivotal intervention, addressing both the patient’s physical and psychological distress, incorporating psychopharmacology, pain management, and psychosocial interventions to effectively restore function and quality of life.

**Disclosure of Interest:**

None Declared

